# Adult revision surgery of prior hook-and-rod wire instrumentation for idiopathic scoliosis

**DOI:** 10.3171/2020.1.FocusVid.19742

**Published:** 2020-01-01

**Authors:** Rebecca M. Burke, Thomas J. Buell, Dominic M. Maggio, Ulas Yener, Chun-Po Yen, Christopher I. Shaffrey, Justin S. Smith

**Affiliations:** 1 Department of Neurological Surgery, University of Virginia Health System, Charlottesville, Virginia; and; 2 Departments of Neurosurgery and Orthopedic Surgery, Duke University Medical Center, Durham, North Carolina

**Keywords:** adjacent-level disease, adult spinal deformity, idiopathic scoliosis, revision spine surgery, ultrasonic bone scalpel, video

## Abstract

Adolescent idiopathic scoliosis patients treated with spinal fusion may develop adjacent segment disease and curve progression into adulthood. Revision operations can be challenging, especially for adult patients treated with outdated instrumentation such as sublaminar hooks and/or wires. The authors demonstrate revision lumbar spine surgery in a 38-year-old female with scoliosis progression from junctional degeneration below a prior T5–L3 posterior instrumented arthrodesis with a hook-and-rod wire system. They also demonstrate safe application of an ultrasonic bone scalpel for completion of a Smith-Petersen osteotomy. The patient provided written, informed consent for all material presented in this case demonstration.

The video can be found here: https://youtu.be/3PmaFtNcqKc.

## Transcript

In this video, we demonstrate a case of revision surgery of an adult patient that had been surgically treated as an adolescent for idiopathic scoliosis. A 38-year-old female with idiopathic scoliosis previously treated with a hook-and-rod wire system in 1996 presented with back and leg pain. Physical exam demonstrated full strength and sensation without evidence of myelopathy. We obtained standing scoliosis X-rays and a CT myelogram. Scoliosis films demonstrate prior instrumentation from T5–L3 with a hook-and-rod wire system. Note the left L3 hook appears displaced from the lamina. SVA measured 3.5 cm. Pelvic incidence measured 40°. Pelvic tilt measured 16.3°. Lumbar lordosis measured 20.3°. Thoracic kyphosis measured 21.1°. She had leftward coronal shift of 2.4 cm. She had a right thoracic curve that measured 20° and a left lumbar curve that measured 32.4°. Her CT myelogram demonstrated multilevel degenerative disease most significant at the adjacent distal levels including L3–4 and L4–5 with associated vacuum disc. The surgical plan involved cutting the distal portions of the previous rods at L1–2; placement of pedicle screw instrumentation starting from L3 through S1 and extending down to the ilium; L3–4, L4–5, and L5–S1 Smith-Petersen osteotomies; L3–4, L4–5, and L5–S1 discectomies and transforaminal lumbar interbody fusion with titanium interbody spacers with dimensions as below; placement of rods spanning from side-to-side connectors at L2–3 extending to the bilateral iliac bolts; and placement of a third accessory rod. Finally, arthrodesis from L1 through the sacrum using BMP, iliac bone graft, locally harvested autograft, and allograft.

**2:36** The patient was positioned prone on a Jackson table in slight reverse Trendelenburg to reduce intraocular pressure. Her arms were positioned at 90° with her abdomen hanging free, and her pressure points were padded appropriately.

**2:50** Neuromonitoring leads were placed for motor evoked and somatosensory evoked potentials as well as EMGs. Neuromonitoring is routinely used for our complex spine cases since they typically involve multilevel osteotomies and complex instrumentation. We often experience a significant degree of correction during these cases, and as such neuromonitoring plays a critical role in alerting the surgeon to operative manipulations that can lead to transient or permanent neurologic deficits.

**3:21** Intraoperative fluoroscopy was used to mark the midline incision to allow access from approximately T12 to the ilium. She was prepped and draped in the usual sterile fashion.

**3:33** A standard posterior exposure of the thoracolumbar and sacral spine was performed which exposed her prior bony fusion mass and instrumentation. The sublaminar hooks and crosslinks distal to the L1–2 level were removed using a combination of the high-speed drill and Leksells. Sagittal benders were used to lift the rod while the assistant removed the hook. The sublaminar wires were cut at these levels but not removed in an effort to avoid a durotomy. In situ cutting of the distal portions of the previous rods at L1–2 was performed.

**4:19** Transpedicular instrumentation was performed from L3 to S1 with iliac screws placed bilaterally. Smith-Petersen osteotomies were performed at L3–4, L4–5, and L5–S1. For educational purposes, we are demonstrating an SPO from an alternative case using an ultrasonic bone scalpel to enhance the educational value of this video.

**4:40** This portion of the video will now be dictated by my colleague Dr. Thomas Buell.

**4:47** First, the interspinous ligament is removed above and below the index level. Then the supraspinous ligament is removed and the spinous process is taken down. The matchstick drill is used to make the initial cut across bilateral pars and lamina. Medial facetectomies are performed. We demonstrate utilization of the ultrasonic bone scalpel to complete the initial cut through the bilateral pars and lamina. Benefits of the bone scalpel include soft tissue preservation with decreased risk of durotomy and decreased bony destruction during the Smith-Petersen osteotomy for increased local autograft for subsequent arthrodesis. Gentle pressure with the bone scalpel should be applied orthogonal to the bony surface. The surgeon can feel decreased resistance or a slight give as the bone scalpel makes a full-thickness cut through bone. Cobb elevators, a rongeur, and an upgoing curette are used to remove the posterior column en bloc. Ligamentous hypertrophy can be decompressed using Kerrison rongeurs, using caution to protect the thecal sac. The superior articulating process or the roof of the neural foramina will now be removed. The initial cut is made with the matchstick high-speed drill and completed with the ultrasonic bone scalpel. The superior articulating process is removed with a rongeur. Further decompression of the lateral recess can be performed. The Penfield can be used to palpate for adequate decompression. The superior articulating process is now removed on the contralateral side using the same steps. We emphasize that the ultrasonic bone scalpel must be used orthogonal to the bone with gentle forward pressure. A slight giveaway is felt when the full-thickness bony cut is completed. The superior articulating process can now be removed. After exposing the disc space during the Smith-Petersen osteotomy, the disc is incised using the inside knife. Using a combination of pituitary rongeurs and rotating cutters, a discectomy is completed. Endplate scrapers can also be utilized to prepare the TLIF. A cage can now be inserted under fluoroscopic guidance.

**7:38** That concludes our demonstration with the bone scalpel. We performed similar steps on our patient and pick up now with insertion of the cage. BMP was introduced into the disc space. Eight-degree titanium lordotic cages were inserted into the disc space at the L3–4, L4–5, and L5–S1 level. The disc space was packed with cancellous bone and the distraction was released. Intraoperative fluoroscopy demonstrated appropriate placement of the instrumentation and cages.

**8:14** Six-millimeter cobalt chromium rods were custom contoured by the surgeon for targeted alignment and placed as seen below. For illustrative purposes, note the accessory rod extending from the right iliac screw to the left original rod.

**8:29** Coronal and sagittal in situ benders were used to further contour the rods to provide targeted alignment. Of note, this was performed prior to the aforementioned step of placement of the accessory rod. Arthrodesis was performed and the incision was closed in a standard layered fashion. Postoperative SVA measured 3.4 cm. Pelvic tilt measured 11°. Lumbar lordosis measured 43.3°. Pelvic incidence measured 40°. Thoracic kyphosis measured 25°. There was significant improvement in her right thoracic and left lumbar curves.

**9:11** Her preoperative back and leg pain improved in the early postoperative period. Her postoperative hospital course was uncomplicated, and she was discharged to home on postoperative day 6. The improvement was sustained throughout her early clinical follow-up.
